# Four Distinct Cases of Multisystem Inflammatory Syndrome in Adults Associated With SARS-CoV-2 Infection at a Community Hospital in New Jersey

**DOI:** 10.7759/cureus.20651

**Published:** 2021-12-23

**Authors:** Charmee Vyas, Denise Dalmacion, Abdalrahman Almeligy, Rojas Juan, Julio D Pernia-Cuberos, Asef Obaid, Farah Heis, Shailee Patel, Margaret H Eng, Chandler D Patton, Andrew Lee

**Affiliations:** 1 Internal Medicine, Monmouth Medical Center, Long Branch, USA; 2 Internal Medicine, Monmouth Medical, East Orange, USA; 3 Critical Care Medicine, RWJBarnabas Health, Long Branch, USA; 4 Infectious Disease, Monmouth Medical Center, Long Branch, USA

**Keywords:** multisystem inflammatory syndrome in adult, sars-cov-2 (severe acute respiratory syndrome coronavirus -2), covid-19, dermatological manifestaion, post-acute covid-19 syndrome

## Abstract

Multisystem inflammatory syndrome (MIS) is a rare entity that usually presents with a constellation of symptoms such as fever, hypotension, gastrointestinal symptoms, cardiac dysfunction, or dermatological involvement, representing an inflammatory state. During the ongoing severe acute respiratory syndrome coronavirus 2 (SARS-CoV-2) pandemic, several cases of multisystem inflammatory syndrome in children (MIS-C) have been described in the literature. The Centers for Disease Control and Prevention (CDC) has acknowledged the increasing incidence of the same entity in adults, referred to as multisystem inflammatory syndrome in adults (MIS-A). This case series describes four patients who presented to the Monmouth Medical Center in New Jersey with symptoms suggestive of MIS-A associated with SARS-CoV-2 infection and their clinical outcomes. All patients were within the age group of 20-40 years with no underlying medical condition. The period between SARS-CoV-2 infection and the development of MIS-A varied from 10 days through a month. Presentations ranged from a mild flu-like illness to shock requiring vasopressors. A positive SARS-CoV-2 antibody test was essential for the diagnosis. Inflammatory markers, such as ferritin, D-dimer, C-reactive protein (CRP), erythrocyte sedimentation rate (ESR), and interleukin-6 (IL-6), were elevated on admission. The Use of immunomodulatory agents, namely steroids and intravenous immunoglobulin (IVIG), resulted in positive clinical outcomes. Inflammatory markers and imaging on admission did not appear to predict the disease course. A positive SARS-CoV-2 polymerase chain reaction (PCR) did not appear to influence the response to treatment. Given the high probability of MIS-A with negative viral testing, the use of both antibody and viral testing with the addition of inflammatory markers may be essential to diagnose this SARS-CoV-2-associated condition.

## Introduction

As per the Centers for Disease Control and Prevention (CDC), multisystem inflammatory syndrome in adults (MIS-A) is defined as a fever that fulfills three clinical criteria, one of which is either severe cardiac illness or rash and non-purulent conjunctivitis. Other criteria include neurological involvement, hypotension, gastrointestinal symptoms, and thrombocytopenia. It is associated with elevated inflammatory markers and a positive severe acute respiratory syndrome coronavirus 2 (SARS-CoV-2) test. During the ongoing SARS-CoV-2 pandemic, several cases of multisystem inflammatory syndrome in children (MIS-C) have been described in the literature. More recently, the CDC has acknowledged the increasing incidence of the same entity in adults: MIS-A. The working definition of MIS-A in current case reports includes the following criteria: (1) severe illness requiring hospital admission in a person aged 21 years or above; (2) a positive test result for either current or previous coronavirus disease 2019 (COVID-19) infection in the past 12 weeks; (3) severe dysfunction of one or more extrapulmonary organ systems; (4) elevated inflammatory markers [e.g., ferritin, C-reactive protein (CRP), D-dimer, interleukin-6 (IL-6)]; and (5) absence of severe respiratory illness [1]. These patients might not have persistent SARS-CoV-2 PCR or antigen positivity, and antibody testing may be necessary to confirm previous SARS-CoV-2 infection. This case series describes four patients who presented to the Monmouth Medical Center during the period from March to June 2021 with symptoms suggesting MIS-A associated with SARS-CoV-2 infection, as well as the treatment plans and their clinical outcomes.

## Case presentation

Case 1

A 38-year-old African American female, with no known medical history, presented with complaints of rash, persistent fevers, and generalized weakness. She had developed a maculopapular rash two days ago that had appeared initially on her thighs; it had eventually progressed to her trunk, upper extremities, and face. She had persistent fevers, with a T-max of 103 °F at home, which prompted her to visit the emergency department (ED). She denied joint or muscle aches, sick contacts, or any recent travel. She denied having had the COVID-19 infection or receiving any COVID-19 vaccine.

On admission, the patient was noted to have high-grade fever (temperature of 103.1 °F), sinus tachycardia (110-120 mmHg), normal blood pressure, and normal pulse oximetry on room air. Her physical exam was significant for bilateral conjunctival injection, mild swelling, and tenderness of the hands, as well as a diffuse maculopapular, non-blanching rash. Chest and abdominal exams were unremarkable.

Initial lab results were significant for mild leukocytosis. Viral/tick-borne illness was suspected and labs were sent accordingly. Inflammatory markers were noticeably elevated: CRP: 201 mg/L (normal level: <7 mg/l), ferritin 468 ng/ml (10-291 ng/ml), and D-dimer 6.292 FEU (0-0.5 FEU). The chest radiograph was unremarkable. Blood, urine, and sputum cultures, as well as rapid streptococcus test, were negative. Initial SARS-CoV-2 PCR result was negative and additional labs, including respiratory viral panel, mononucleosis, HIV, syphilis, hepatitis panel, cytomegalovirus, Rocky Mountain spotted fever, West Nile virus, and antinuclear antibodies (patient’s sister had lupus), were negative. She was treated symptomatically, pending lab results on the day of admission; however, the very next day, she left against medical advice. She had a repeat ED visit a day later due to worsening symptoms. A second PCR test was positive, but she was also noted to have SARS-CoV-2 IgG antibodies at this time.

She was started on intravenous (IV) steroids for suspected MIS-A and systemic anticoagulation for the hypercoagulable state. Despite treatment, her fever persisted. She subsequently developed diarrhea and tested negative for Clostridioides difficile (C. difficile) and other stool studies. On the third day of hospitalization, the rash improved, her diarrhea resolved, and she remained afebrile for 48 hours. She was discharged on oral methylprednisolone taper and apixaban due to the persistently elevated D-dimer levels throughout her admission.

Case 2

A 24-year-old African American male with no significant past medical history experienced mild symptomatic COVID-19 infection that resolved with conservative management four weeks before admission. Thereafter, he had headaches, abdominal pain, nausea, and diarrhea starting nine days before admission when he was retested for SARS-CoV-2 PCR but found to be negative. He was managed as a case of viral gastroenteritis. Two days before admission, he had started to have progressively worsening shortness of breath, which had prompted his ED visit. 

Initial vital signs were notable for blood pressure in the 70s/40s, heart rate in the 120s, respiratory rate in the 40s, and a high-grade fever of 102.6 °F. Oxygen saturation was 93% on ambient air. The initial physical exam was notable for lethargy and a petechial rash involving both legs. He was tachycardic, had regular rhythm, no murmur, his lungs were clear to auscultate, and there was no abdominal tenderness.

Initial labs were significant for a white blood cell count of 26 with bandemia and elevated inflammatory markers: ferritin: 1294 ng/ml (10-322 ng/ml), erythrocyte sedimentation rate (ESR): 67 mm/hr (0-15 mm/hr), CRP: 268 mg/L (<7 mg/l). Troponin was elevated at 1.077 ng/ml; brain natriuretic peptide (BNP) level was 1206 pg/ml, and lactic acid of 2.4 mmol/L was noted. Chest X-ray demonstrated multifocal consolidations bilaterally. SARS-CoV-2 PCR was positive on admission and SARS-CoV-2 IgG was also positive. Electrocardiography revealed sinus tachycardia, normal axis, with no ST-segment or T wave abnormalities. CT angiography of the chest was negative for pulmonary embolism.

The initial impression was of sepsis from viral/superadded bacterial pneumonia. He received broad-spectrum antibiotics - vancomycin and cefepime - in the ED after cultures were sent. He also received saline 30 cc/kg but his blood pressure continued to be low. He had to be started on vasopressors due to persistent hypotension. Given his recent COVID-19 infection and extrapulmonary involvement (circulatory system), MIS-A was considered and the patient was given IV immunoglobulin (IVIG) and high-dose IV steroids (IV methylprednisone 125 mg every six hours). The next day, his mental and respiratory status improved and he was given another dose of IVIG. Bedside point-of-care cardiac ultrasound revealed decreased left ventricular ejection fraction of 35-40%, with no left ventricular dilation, and a diagnosis of myocarditis was made. He was continued on antibiotics and high-dose steroids. He required vasopressor support for four days before he was successfully weaned off. He was continued on steroids and when the repeat echo showed an improved ejection fraction of 50-55%, steroid taper was started. Over the course of the next few days, his condition improved and inflammatory markers tapered. He was discharged on a prednisone taper for two weeks. Thereafter, he was seen in the clinic with complete resolution of symptoms.

Case 3

A 39-year-old Caucasian male, with no significant medical history, presented with complaints of fever, chills, and night sweats of one-week duration. He had also been having nasal congestion, headache, and fatigue for approximately the same duration. He had been tested for SARS-CoV-2 PCR twice, the first time as an outpatient about a week before admission, and the second time at admission, which had returned negative. He complained of bouts of cough and mediastinal discomfort on deep inhalation and palpitations on minimal exertion. He had completed a five-day course of azithromycin as an outpatient without improvement in his symptoms. On physical exam, he appeared comfortable and was essentially unremarkable. He had a papule on his right forearm that had the appearance of an insect bite.

Initial labs revealed elevated inflammatory markers: ferritin: 847.3 ng/ml (10-322 ng/ml), D-dimer: 0.25 FEU (0-0.5 FEU), lactate dehydrogenase (LDH): 283 IU/L (116-243 IU/L), CRP: 35.5 mg/L (<7 mg/l), and IL-6: 35.9 pg/ml (<5 pg/ml). Peripheral smear was negative for malaria. Chest radiograph showed multifocal consolidations of the right lung. He received a five-day remdesivir course and dexamethasone due to initial suspicion for acute SARS-CoV-2 infection. A SARS-CoV-2 PCR was repeated and came back negative, and the SARS-CoV-2 IgG antibody came back positive. Lyme IgG antibody, Legionella urine antigen, Strep pneumococcal urine antigen, and urine and blood cultures came back negative. The patient was eventually managed as a case of MIS-A and was discharged on a low dose of Decadron for five days.

Case 4

A 20-year-old female had mild upper respiratory tract symptoms due to COVID-19 and was managed conservatively. She was admitted a month and a half later to the psychiatry unit with newly diagnosed depression with suicidal ideation. Her SARS-CoV-2 PCR on admission was negative. She was asymptomatic at that time with an unremarkable physical exam. After the first 24 hours, she developed diarrhea and was managed symptomatically. On the third day of admission, she started spiking fevers with a T-max of 103.9 °F, was tachycardic in the 130s, but saturating well on room air. A repeat SARS-CoV-2 PCR test was conducted, which was also negative. She was transferred to the medicine service and workup was ordered for acute febrile illness.

The diagnostic workup was unremarkable. Chest radiograph did not show any infiltrates and cultures were all negative. Her lactic acid was elevated at 2.1 mmol/L, and she was started on IV fluids. A respiratory panel was negative and procalcitonin was 0.1 ng/ml. The patient continued to have a fever with chills, sore throat, and multiple loose bowel movements. She had elevated CRP with a peak of 164.67 mg/l (<7 mg/l). Her COVID-19 IgG was positive. The patient was conservatively managed and was discharged after the complete resolution of her symptoms.

The night after discharge, she woke up with substernal pressure radiating to the back, associated with diaphoresis, prompting her to come back to the ED. On readmission, her EKG showed ST elevations in V5, V6 with elevated troponin at 13.021 ng/ml. The patient was lymphopenic and her inflammatory markers were elevated (D-dimer and CRP). The echocardiogram was unremarkable. The patient was admitted with the diagnosis of MIS-A myocarditis and was treated with colchicine. Her symptoms improved the next day and she did not require steroids. She was eventually discharged.

**Table 1:** Demographics, clinical features, treatments, and outcomes of four adults with multisystem inflammatory syndrome associated with SARS-CoV-2 infection Ab: antibody; CRP: C-reactive protein; CT: computed tomography; CXR: chest X-ray; GI: gastrointestinal; IVIG: intravenous immunoglobulin; PE: pulmonary embolism; SARS-CoV-2: severe acute respiratory syndrome coronavirus 2

	Patient 1	Patient 2	Patient 3	Patient 4
Demographics				
Age in years, gender	38, female	24, male	39, male	20, female
Race/ethnicity	African American	African American	Caucasian	Caucasian
Medical history	None	Vaping or e-cigarette use	None	Major depressive disorder
Clinical presentation				
Duration from symptom onset to admission, days	2	9	7	6
Initial symptoms	Rash, fever, weakness	Shortness of breath, GI symptoms, headache	Fever, night sweats, fatigue	Diarrhea, fever, chest pain, fatigue, sweating
Laboratory results				
Peak CRP, mg/L	206.68	268.95	54.64	164.76
Peak ferritin, ng/mL	582	1645.8	1200.4	208.5
Peak d-dimer, ug/mL	6.292	1.863	0.422	0.605
IL-6, pg/mL	No	No	35.91	No
SARS-CoV-2 PCR	1st: negative, 2nd: positive	1st: negative, 2nd: positive	Negative	Negative
SARS-CoV-2 Ab	Positive	Positive	Positive	Positive
Imaging				
CXR/CT	CXR: unremarkable	CXR: multifocal consolidations, bilateral; CT: no PE, ground-glass opacities bilaterally, small bilateral pleural effusions	CXR: multifocal consolidations of the right lung	CXR: unremarkable
Treatment				
Anti-inflammatory drugs	Methylprednisolone 1 mg/kg/day x 2 days, taper	Methylprednisolone 1 mg/kg/day x 7 days, taper	Dexamethasone x 5 days	Colchicine
IVIG	No	2 mg/kg for 2 days	2 mg/kg x 1 day	None
Remdesivir	No	No	Yes	None
Vasopressor use	None	Norepinephrine	None	None
Outcome	Discharged	Discharged	Discharged	Discharged
Length of hospital stay, days	3	7	6	6

## Discussion

While MIS-C has become a recognized pathology, the same entity in adults has yet to be fully described. MIS-C was first reported in April 2020 as a hyperinflammatory syndrome with features that resembled Kawasaki’s disease, with varied clinical signs and symptoms but predominantly including shock, cardiac dysfunction, abdominal pain, mucocutaneous manifestation, with laboratory findings of elevated inflammatory markers such as CRP, ferritin, D-dimer, and IL-6. Several case reports from the United Kingdom and the United States from March through December 2020 have described a hyperinflammatory state associated with SARS-CoV-2 in adults with features similar to MIS-C [1].

This case series described four patients aged 20-39 years, who presented during the period from March to June 2021 with clinical and laboratory findings consistent with MIS-A associated with SARS-CoV-2 infection in a small community hospital in New Jersey, United States. All patients had laboratory confirmation of a concurrent or previous history of SARS-CoV-2 infection. Two of the patients tested positive for SARS-CoV-2 PCR but all patients had detectable antibodies by serologic assay at the time of presentation. The time period between infection and the development of MIS-A was unknown. Antibodies usually develop within two weeks of infection [2]. It varied from 10 days through one month after the onset of typical COVID-19 symptoms in our patients.

Presentations varied from a mild flu-like illness to shock requiring vasopressors. Fever was the most common symptom. In three patients, cardiovascular involvement was present. All patients were young and had no underlying medical condition. Peak inflammatory markers as well as the severity of imaging on admission did not appear to predict the disease course or clinical outcomes. A positive SARS-CoV-2 PCR did not appear to influence response to treatment. In our series, immunomodulatory agents (methylprednisolone, dexamethasone, and IVIG) resulted in positive clinical outcomes. Concomitant treatment with remdesivir did not appear to alter the response to steroids. All patients had good clinical outcomes and were discharged from the hospital on steroid taper. Length of stay ranged from three to seven days.

The pathophysiology and mechanism of the MIS in both children and adults are unknown. The presence of antibodies often portends protection. However, pathogen-specific antibodies can lead to antibody-dependent enhancement, resulting in pathology, such as those seen in the dengue virus. It has been demonstrated in patients infected with SARS-CoV that early seroconversion and higher IgG titers correlate with disease severity [3,4]. SARS-CoV and SARS-CoV-2 share similar sequences and utilize the same receptor for entry, and hence SARS-CoV-2 antibodies may undergo the same mechanism. It has been postulated that pathogenesis may result from the inflammatory cascade and viral entry enhancement of non-neutralizing antibodies in SARS-CoV-2 [3]. Several factors, such as specificity, concentration, affinity, and isotype of the antibody, determine the role of antibodies.

Further research is necessary to inform clinical management and prevention efforts. Given the high probability of MIS-A with negative viral testing, the use of both antibody and viral testing with the addition of a laboratory panel for inflammation, hypercoagulability, and organ damage (e.g., CRP, ESR, ferritin, liver enzymes, D-dimer) may be essential for the early recognition of this SARS-CoV-2-associated condition. The mechanism of antibody-mediated immunity in SARS-CoV-2 is currently unknown. A comprehensive assay may be necessary to profile the humoral response among previously infected patients. The lack of clear clinical guidance regarding MIS-A highlights the importance of conducting further research to establish a standardized algorithm for diagnosis.

## Conclusions

This case series elaborated on four different cases of MIS-A. SARS-CoV-2 is a relatively novel disease, and we continue to learn about its various short and long-term sequelae. MIS-A can have various outcomes, ranging from benign to potentially fatal, and hence it is crucial to have it as a differential diagnosis for someone presenting to the hospital within a month of COVID-19 infection. A positive SARS-CoV-2 IgG antibody with elevated inflammatory markers can assist with diagnosing MIS-A. While the precise mechanism of MIS-A remains to be understood, our literature review has consistently indicated that antibody-dependent enhancement might be the causal mechanism. Three of the four patients in our case series were administered systemic steroids and showed remarkable improvement. Steroids and IVIG are currently being used as the first-line treatment; however, further research is necessary to define the exact role of the same in the recovery process. This case series is limited by the lack of long-term follow-up on our patients; however, it still sheds crucial light on the varied presentation of MIS-A and its successful treatment.

## References

[1] Morris SB, Schwartz NG, Patel P (2020). Case series of multisystem inflammatory syndrome in adults associated with SARS-CoV-2 infection - United Kingdom and United States, March-August 2020. MMWR Morb Mortal Wkly Rep.

[2] Wang K, Long QX, Deng HJ (2021). Longitudinal dynamics of the neutralizing antibody response to severe acute respiratory syndrome coronavirus 2 (SARS-CoV-2) infection. Clin Infect Dis.

[3] Iwasaki A, Yang Y (2020). The potential danger of suboptimal antibody responses in COVID-19. Nat Rev Immunol.

[4] Lee N, Chan PK, Ip M (2006). Anti-SARS-CoV IgG response in relation to disease severity of severe acute respiratory syndrome. J Clin Virol.

